# Hyperoxia is Dose-Dependently Associated with an Increase of Unfavorable Outcomes in Ventilated Patients with Aneurysmal Subarachnoid Hemorrhage: A Retrospective Cohort Study

**DOI:** 10.1007/s12028-022-01534-y

**Published:** 2022-06-08

**Authors:** Jörn Grensemann, Marius Marc-Daniel Mader, Manfred Westphal, Stefan Kluge, Patrick Czorlich

**Affiliations:** 1 Department of Intensive Care Medicine, University Medical Center Hamburg-Eppendorf, Martinistraße 52, 20246 Hamburg, Germany; 2 Department of Neurosurgery, University Medical Center Hamburg-Eppendorf, Martinistraße 52, 20246 Hamburg, Germany; 3 Institute for Stem Cell Biology and Regenerative Medicine, Stanford University School of Medicine, Stanford, CA USA

**Keywords:** Subarachnoid hemorrhage, Mechanical ventilation, Hyperoxia, Hyperoxemia, Oxygen, Mortality, Outcome

## Abstract

**Background:**

Adequate oxygenation in patients with aneurysmal subarachnoid hemorrhage (SAH) is imperative. However, hyperoxia increases formation of reactive oxygen species and may be associated with a dose-dependent toxicity. We postulated a threshold for arterial partial pressure of oxygen (paO_2_) above which toxicity effects precipitate and sought to study the effects on 30-day mortality, favorable outcome at discharge and at 3 months, and delayed cerebral ischemia.

**Methods:**

In this retrospective single-center cohort study, patients with SAH and mechanical ventilation > 72 h were included. Oxygen integrals were calculated above the following thresholds: 80, 100, 120, and 150 mm Hg and time-weighted mean paO_2_. All calculations were done from admission to end of day 1, day 3, and day 14. We conducted multivariable logistic regression analyses adjusted for age, sex, duration of ventilation, and Hunt and Hess grade. Time-weighted mean paO_2_ was categorized by quartiles. Favorable outcome was defined as Glasgow Outcome Scale scores of 4 and 5.

**Results:**

From November 2010 to February 2021, 282 of 549 patients fulfilled the inclusion criteria. Odds ratios for 30-day mortality increased dose dependently and were as follows: 1.07 (95% confidence interval [CI] 1.03–1.11; *p* = 0.001) for each 1 mm Hg per day above 80 mm Hg; 1.16 (95% CI 1.07–1.27), above 100 mm Hg; 1.36 (95% CI 1.15–1.61), above 120 mm Hg; and 1.59 (95% CI 1.22–2.08), above 150 mm Hg (all *p* < 0.001) at day 14. For favorable outcome at 3 months, odds ratios were 0.96 (95% CI 0.92–0.99) for each 1 mm Hg per day above 80 mm Hg; 0.90 (95% CI 0.84–0.98), above 100 mm Hg; 0.83 (95% CI 0.72–0.97), above 120 mm Hg; and 0.77 (95% CI 0.61–0.97), above 150 mm Hg (all *p* < 0.05). For time-weighted mean paO_2_, lowest 30-day mortality and highest favorable outcome at 3 months were found in the second quartile (78–85 mm Hg). Thirty-day mortality increased above 93 mm Hg (fourth quartile), with an odds ratio of 3.4 (95% CI 1.4–8.4, *p* = 0.007). Odds ratios for favorable outcome at 3 months were 0.28 (95% CI 0.12–0.69), 0.27 (95% CI 0.11–0.67), and 0.24 (95% CI 0.10–0.59) for the first, third, and fourth quartiles, respectively (all *p* < 0.01). No significant association was found at day 1 and day 3, for favorable outcome at discharge, or for delayed cerebral ischemia.

**Conclusions:**

Integrals above the defined paO_2_ thresholds were dose-dependently associated with an increase in mortality in ventilated patients with SAH. When we considered time-weighted mean paO_2_, unfavorable outcomes and 30-day mortality were more frequent both below and above a certain range. Unfavorable outcomes increased in paO_2_ ranges usually defined as normoxia. This emphasizes the necessity to further characterize oxygenation thresholds in ventilated patients with SAH in prospective clinical studies.

**Supplementary Information:**

The online version contains supplementary material available at 10.1007/s12028-022-01534-y.

## Introduction

Aneurysmal subarachnoid hemorrhage (SAH) leads to a significant disease burden, with many patients remaining disabled [1, 2]. In the acute phase of SAH, oxygenation of brain tissue is paramount, particularly in the event of vasospasms or delayed cerebral ischemia (DCI) [3–5]. However, increasing oxygen delivery leads to the formation of reactive oxygen species (ROS), which may induce apoptosis or necrosis and worsen outcome [6–8]. Recent data indicate that hyperoxia, defined as exposure of tissue to supranormal partial pressures of oxygen, within the first 24 h after admission increases the risk for DCI [9, 10] and worsens outcome, particularly in patients with poor-grade SAH [11]. These studies evaluated peak, mean, or time-weighted oxygen partial pressures. Oxygen is essential in the respiratory chain for aerobic metabolism, but increasing mitochondrial oxygen partial pressures does not increase the metabolic rate but may induce mitochondrial damage [12]. Therefore, we hypothesized the existence of an arterial partial pressure of oxygen (paO_2_) threshold at which sufficient oxygen pressures are maintained for aerobic metabolism, but formation of ROS is still negligible. On the basis of the hypothesis of a dose-dependent oxygen toxicity above this threshold, we aimed to identify an optimum target range for mechanically ventilated patients with SAH.

## Methods

### Ethics

The retrospective and anonymized data collection and analysis were conducted in accordance with local government law (HmbKHG. §12) without the requirement for approval or informed consent. The study was performed in accordance with the ethical standards as laid down in the 1964 Declaration of Helsinki and its later amendments or comparable ethical standards.

### Study Design

This study was a retrospective, single-center, exploratory cohort study.

### Setting and Population

The study was jointly conducted at the Department of Intensive Care and Department of Neurosurgery at University Medical Center Hamburg-Eppendorf. Patients were included if they were admitted for aneurysmal SAH confirmed by digital subtraction angiography, computer tomography angiography, or magnetic resonance angiography and mechanically ventilated for at least 72 h. Patients were included since the introduction of electronic patient records in November 2010 until February 2021.

### Outcome Parameters

**Fig. 1:**
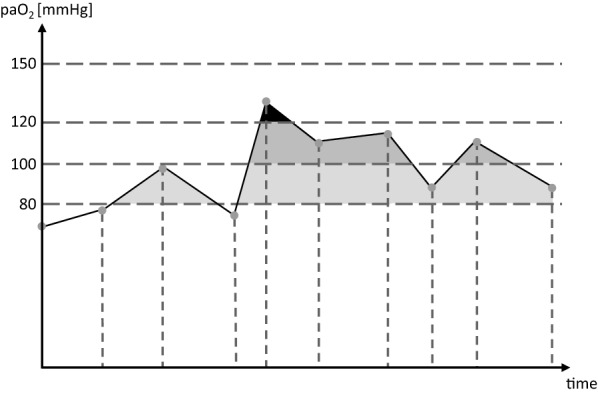
Calculation of integrals. Exemplary calculation of integrals above arbitrarily chosen limits; horizontal dotted lines depict limits above which the respective integrals are calculated; gray dots and vertical dotted lines depict arterial oxygen partial pressures obtained by arterial blood gas analyses (integral above 80 mm Hg: light gray + medium gray + black shaded areas; integral above 100 mm Hg: medium gray + black shaded areas; integral above 120 mm Hg: black shaded area; integral above 150 mm Hg is zero). paO_2_ arterial oxygen partial pressure

The influence of oxygenation on 30-day mortality, outcome assessed by the Glasgow Outcome Scale (GOS) at discharge and at 3 months, and DCI were evaluated. Favorable outcome was defined as a GOS score of 4 or 5. DCI was defined according to criteria published by Vergouwen et al. [13]. The following oxygenation parameters were calculated: paO_2_ integrals above thresholds of 80, 100, 120, and 150 mm Hg. The thresholds of 100, 120, and 150 mm Hg were adopted from previous studies [11, 14], and 80 mm Hg was included to differentiate the range below 100 mm Hg more precisely given the lack of information in the literature. A graphical depiction of calculations is given in Fig. 1. Time-weighted mean paO_2_ was calculated, and the maximum paO_2_ was obtained. Additionally, time-weighted mean paO_2_ was categorized according to quartiles to determine optimum target ranges. All calculations were done (1) for the first 24 h after admission as the hyperacute phase, (2) from admission up to day 3 as the acute phase, and (3) from admission up to day 14. For the calculation of integrals, linear changes between paO_2_ values obtained from the arterial blood gas (ABG) analyses were assumed. ABG values were obtained at least every 4 h. To allow for comparisons, the integrals for up to days 3 and 14 were calculated as mean integrals per day. For patients who died before the end of the observation period, calculations were performed using the available data. All calculations of oxygenation parameters were done with Visual Basic for Applications (V7.1; Microsoft Corp., Redmond, WA).

### Data Retrieval

Data were extracted from the electronic intensive care patient data management system (Intensive Care Manager, V10; Drägerwerk, Lübeck, Germany) with the ICMiq data extraction tool (V1.3; Drägerwerk). We obtained demographic and descriptive data and data on mechanical ventilation and ABG. Data management was done with Microsoft Excel 2019 (Microsoft Corp.).

### Statistics

Statistical analyses were performed using SPSS (version 27; IBM Corp., Armonk, NY). We used binary multivariable logistic regression analyses for the dependent variables 30-day mortality, favorable outcome at discharge and 3 months, and DCI. Separate models were calculated for different oxygenation parameters as independent variables. Age, Hunt and Hess grade, sex, and total duration of mechanical ventilation were a priori selected as covariates.

## Results

From November 2010 to February 2021, 549 patients with SAH were identified. From these, 282 patients were ventilated for more than 72 h (Supplementary Fig. S1). An overview on patients’ characteristics is provided in Table 1. Data on discontinuation of mechanical ventilation before day 14 are provided in the supplement (Supplementary Fig. S3, Supplementary Table S10). For the analysis of outcome at 3 months, 22 patients were lost to follow-up. Of the included patients, 68% were female, and the 30-day mortality rate was 26%. The disease severity characterized by the Hunt and Hess grade was as follows: 1, 11%; 2, 16%; 3, 20%; 4, 21%; and 5, 32%.

**Table 1:** Patient characteristics

Patient characteristics	*N* = 282
Age (years)	56.9 ± 13.7
Weight (kg)	76.7 ± 15.4
Height (cm)	171.3 ± 14.3
Sex	
Female	193 (68%)
Male	89 (32%)
Hunt and Hess grade	
1	32 (11%)
2	46 (16%)
3	56 (20%)
4	58 (21%)
5	90 (32%)
Fisher grade	
1	9 (3%)
2	13 (5%)
3	39 (14%)
4	220 (78%)
Missing	1
Glasgow Coma Scale score	
13–15	123 (44%)
9–12	21 (7%)
4–9	57 (20%)
3	68 (24%)
Missing	13 (5%)
DCI	117 (42%)
Aneurysm localization	
Anterior circulation	210 (74%)
Posterior circulation	72 (26%)
Treatment	
Microsurgical clipping	90 (32%)
Endovascular coiling	173 (62%)
Other (e.g., flow diverter)	19 (6%)
Rebleeding	58 (21%)
Acute hydrocephalus	231 (82%)
Ventriculoperitoneal shunting	58 (21%)
Seizure	64 (23%)
Mortality	73 (26%)
Length of ventilation (days)	14.3 ± 11.5

Data are given as mean ± standard deviation or numbers with percentages in parentheses, as applicable

*DCI* delayed cerebral ischemia

**Fig. 2:**
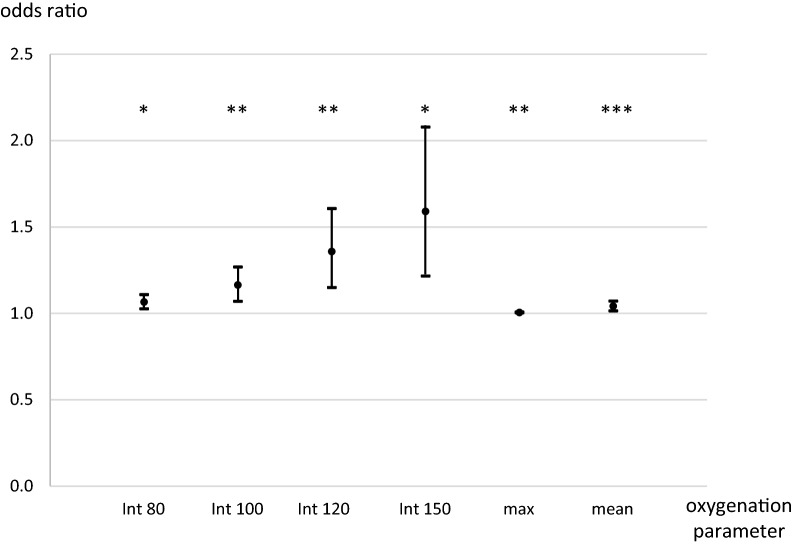
Odds ratios for 30-day mortality for hyperoxia over 14 days. Int: arterial oxygen partial pressure integral above given threshold (mm Hg). Odds ratios for 30-day mortality for the integrals depicting a mean increase of 1 mm Hg/day for 14 days above the given threshold. Max: maximum arterial oxygen partial pressure in mm Hg for 14 days. Mean: mean arterial oxygen partial pressure in mm Hg, odds ratio for an increase of 1 mm Hg/day for 14 days. Separate models were calculated for each oxygenation parameter. *p = 0.001; **p < 0.001; ***p = 0.003 (all values versus baseline). Error bars indicate 95% confidence intervals

Concerning 30-day mortality, the results of the univariate analysis are shown in Supplementary Table S1. In the multivariable logistic regression analysis adjusted for age, sex, Hunt and Hess grade, and length of ventilation, all calculated integrals and the mean and maximum paO_2_ over day 14 showed a significant influence on 30-day mortality. Increased odds ratios for 30-day mortality were observed with higher paO_2_ integrals (Fig. 2, Supplementary Table S5).

**Fig. 3:**
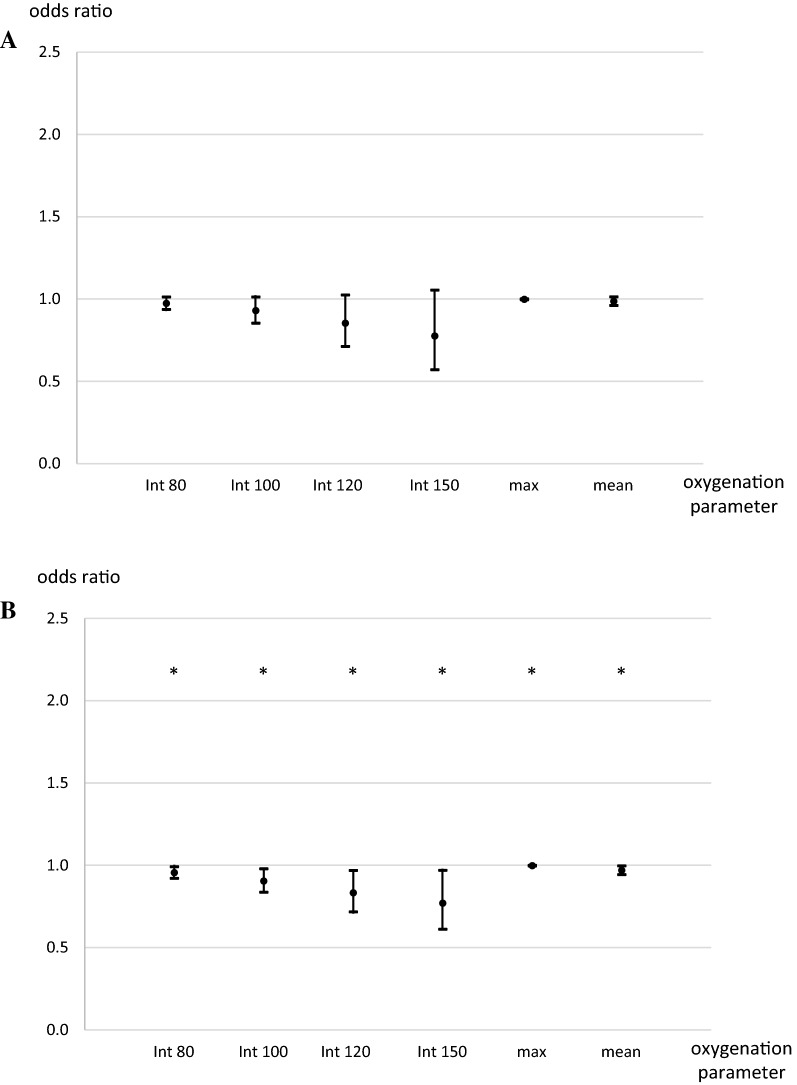
Odds ratios for favorable outcome for hyperoxia over 14 days. **a** At discharge. **b** At 3 months. Int: arterial oxygen partial pressure integral above given threshold (mm Hg). Odds ratios for 30-day mortality for the integrals depicting a mean increase of 1 mm Hg/day for 14 days above the given threshold. Max: maximum arterial oxygen partial pressure in mm Hg for 14 days. Mean: mean arterial oxygen partial pressure in mm Hg, odds ratio for an increase of 1 mm Hg/day for 14 days. Separate models were calculated for each oxygenation parameter. *p < 0.05 versus baseline. Error bars indicate 95% confidence intervals

For favorable outcome by GOS score at discharge, the univariate analysis revealed significant differences for age, Hunt and Hess grade, and length of ventilation (Supplementary Table S2). In the univariate analysis, integrals and maximum paO_2_ over day 14 were different, but the multivariable logistic regression analysis failed to establish a statistically significant association, although a trend to decrease of favorable outcome with increasing oxygen integrals over day 14 may be observed (Fig. 3a).

For favorable outcome by GOS score at 3 months, the univariate analysis revealed significant differences for age, Hunt and Hess grade, and length of ventilation (Supplementary Table S3). In the multivariable analysis, integrals and mean and maximum paO_2_ with higher oxygenation values over day 14 were associated with a significant decrease of favorable outcomes (Fig. 3b, Supplementary Table S7).

For DCI, the univariate analysis revealed significant differences for Hunt and Hess grade, length of ventilation, and maximum paO_2_ at 14 days within the groups (Supplementary Table S4). No significant influence of any oxygenation parameter could be established between the groups in a multivariable regression model (Supplementary Table S8). Including DCI as a covariate in the multivariable logistic regression analyses of 30-day mortality and favorable outcomes did not relevantly change the models (data not shown).

Time-weighted mean paO_2_ was categorized between the following values: 62 (lowest), 78 (quartile one), 85 (median), 93 (quartile three), and 228 mm Hg (highest). The distribution of time-weighted mean paO_2_ is shown in Supplementary Fig. S2.

**Fig. 4:**
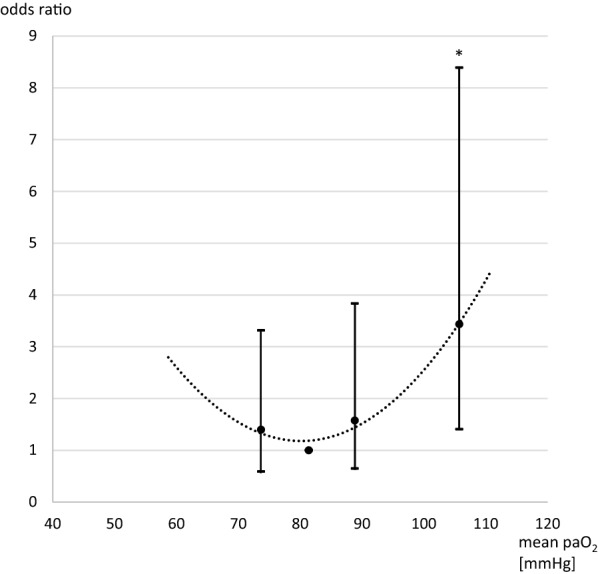
Odds ratios for 30-day mortality and optimum range from categorized time-weighted mean arterial oxygen partial pressures over 14 days. paO_2_ arterial partial pressure of oxygen. Dotted line depicts quadratic trendline. *p = 0.007 versus quartile two. Error bars indicate 95% confidence intervals

Thirty-day mortality was lowest between time-weighted mean paO_2_ values of 78 and 85 mm Hg at 20% and increased to 28% below 78 mm Hg (odds ratio of 1.4 [95% confidence interval (CI) 0.6–3.3]), 23% between 85 and 93 mm Hg (odds ratio 1.6 [95% CI 0.6–3.8]), and 32% above 93 mm Hg (odds ratio 3.4 [95% CI 1.4–8.4], *p* = 0.007) (see Fig. 4).

Favorable outcomes at 3 months were highest between 78 and 85 mm Hg at 53% and decreased to 32% below 78 mm Hg (odds ratio 0.28 [95% CI 0.12–0.69], *p* = 0.006), 39% between 85 and 93 mm Hg (odds ratio 0.27 [95% CI 0.11–0.67], *p* = 0.004), and 32% above 93 mm Hg (odds ratio 0.24 [95% CI 0.10–0.59], *p* = 0.002).

## Discussion

In this retrospective analysis of patients with SAH ventilated for at least 72 h, we could show an association of hyperoxia and 30-day mortality with a dose-dependent effect for more severe hyperoxia. However, 30-day mortality and unfavorable outcomes also increased with lower paO_2_ values. Favorable outcomes decreased with hyperoxia, formally reaching statistical significance at 3 months. No association between oxygenation and DCI could be established.

Providing sufficient oxygen delivery to the brain is imperative in neurocritical care patients. Oxygen delivery depends on various factors, e.g., a sufficient cerebral perfusion pressure, blood flow, and hemoglobin level [3]. Oxygen partial pressures must be adequate as well. However, hyperoxia induces the formation of ROS, promoting cell damage [8, 15]. Moreover, hyperoxia may induce vasoconstriction and a reduction of cerebral blood flow velocities [16]. Further mechanisms leading to negative effects have been proposed, such as direct mitochondrial damage, triggering of neuroinflammation, or blood–brain barrier breakdown, besides extracerebral manifestations, such as pulmonary toxicity or a coronary vasoconstriction [17]. Clinically, oxygen toxicity may trigger neurologic symptoms, e.g., nausea, dizziness, or convulsions [8].

Physiologically, because of its sigmoidal binding curve, hemoglobin binds only little additional oxygen above a paO_2_ of 80 mm Hg, corresponding to an oxygen saturation (SO_2_) of approximately 96%, whereas ROS formation may increase [18]. Current guidelines recommend targeting an SO_2_ of 92–96% in critically ill patients [19] or maintaining an SO_2_ above 94% in patients with stroke [20]. However, there is still a paucity of data concerning optimal oxygenation targets.

To date, no prospective studies have evaluated oxygenation targets specifically for patients with SAH with respect to outcome parameters. Previous retrospective studies indicated negative effects for hyperoxia. Higher paO_2_ values on the first day were dose-dependently associated with poorer outcomes [10, 11, 14, 21]. Furthermore, an association of higher paO_2_ on day 1 with more frequent DCI could be demonstrated [10]. Jeon et al. [9] calculated oxygen burden as paO_2_ integrals over time that equaled time-weighted mean paO_2_ as calculated in our study and could show a decrease of favorable outcomes for higher oxygen burdens. Oxygen burden was assessed until onset of DCI or up to day 6, and an oxygen level above 173 mm Hg was also associated with DCI [9]. Evaluating oxygen exposure up to day 6, Fukuda et al. [10] observed no effect on DCI or outcome by GOS score at a mean paO_2_ of 120 mm Hg in both groups.

As opposed to our patient cohort, patients in these studies had far higher paO_2_ values, and the definition of hyperoxia ranged from a paO_2_ value above 120 mm Hg to a paO_2_ value above 200 mm Hg. None of these studies included a significant quantity of patients with mean paO_2_ values below 100 mm Hg. As outlined above, only little additional oxygen is transported above a paO_2_ of 80 mm Hg, and the formation of ROS and other hyperoxia-induced negative effects may outweigh the benefit of additional oxygen transport above 100 mm Hg [18] or even at lower values, as indicated by our data.

In our hospital, we generally target lower paO_2_ values than have been attained in the studies cited above because of increasing knowledge in recent years about unfavorable effects of hyperoxia [6, 18]. We attribute the missing association between the outcome parameters in our study and oxygenation in the first days of treatment to the lower oxygen burden in our patients. Presumably, a higher oxygen burden was already reached at day 1 in previous studies, leading to the observed toxicity effects. However, it is possible that overall lower mean paO_2_ values in our study population prolonged the time until a critical oxygen burden was reached. Therefore, effects on outcome were only statistically significant in a 14-day observation period including patient data with ventilation over a longer time window. This would be in line with the concept of a dose-dependent toxicity.

In our study, 30-day mortality already increased and favorable outcomes decreased above a time-weighted mean paO_2_ of 85 mm Hg. The paO_2_ range of 78 to 85 mm Hg associated with the most favorable outcomes in our study coincides with the range that may be chosen from a theoretical point of view balancing sufficient oxygenation versus ROS. However, a value of 85 mm Hg is usually considered as normoxic. Therefore, we suggest that prospective studies consider and further examine paO_2_ ranges outlined in this study in the search for the optimum oxygenation target range in patients with SAH. Notably, values below 78 mm Hg were also associated with a decrease of favorable outcomes, leading to a U-shaped curve between hyperoxia and outcome with an optimum oxygen target range. The 78 mm Hg cutoff value is higher than some recommendations on oxygen targets but is congruent to a study showing paO_2_ levels below 80 mm Hg during brain tissue hypoxia [5]. No studies exist that systematically evaluate hypoxia in critically ill patients, but adequate oxygenation is nevertheless crucial, and hypoxia may not be tolerated.

When we reviewed the available data on oxygen targets in patients with SAH, no benefit could be shown for hyperoxia, and some studies could even show harm [22]. However, no hyperoxia threshold or optimum oxygen target range could be established so far. With our study, we identified a range in which a potential hyperoxia threshold and an optimum oxygen target range may be situated, and we deem prospective confirmatory studies necessary, as has been suggested by other authors as well [22]. For example, comparing a target range of 75–85 mm Hg vs. 95–105 mm Hg approximating our quartiles 2 and 4 would require approximately 400 patients to detect a 14% difference in favorable outcome at 3 months.

Our study has certain limitations. With our study design, we could show an association between oxygenation and outcome, which does not translate into causality, but the aim of our study was exploratory and should generate a hypothesis to be evaluated in forthcoming prospective trials. Because of the retrospective design, we cannot exclude that our results were confounded by further variables, but our results are congruent to previously published data. We included only patients ventilated for at least 72 h to increase homogeneity of the studied patients by excluding patients only being ventilated after initial surgery or neuroradiological intervention for aneurysm occlusion. The defined paO_2_ thresholds in this study were arbitrarily chosen, which was attributable to the exploratory design. Furthermore, instances of hyperoxia during preoxygenation, for example, suctioning procedures more prevalent in ventilated patients could not be considered because our calculations were based on ABGs. Hypoxia may increase 30-day mortality too, but sufficient data on periods of hypoxia could not be retrieved from our records. We did not systematically measure regional cerebral oxygenation via brain tissue oxygenation probes.

## Conclusions

In our study, we demonstrate a dose-dependent association between sustained hyperoxia and a higher 30-day mortality as well as reduced favorable functional outcome at 3 months. The time-weighted paO_2_ range of 78–85 mm Hg was associated with the lowest 30-day mortality and highest favorable outcomes. We recommend that further prospective studies evaluate values in this range, which is usually considered as normoxic, in this special patient population.

## Supplementary Information

Below is the link to the electronic supplementary material.

Supplementary file 1 (DOCX 378 kb)
