# Supplementary material for: Hyperoxia is Dose-Dependently Associated with an Increase of Unfavorable Outcomes in Ventilated Patients with Aneurysmal Subarachnoid Hemorrhage: A Retrospective Cohort Study
Source: Neurocrit Care. 2022 Jun 8;37(2):523–30. doi: 10.1007/s12028-022-01534-y (PMC9519732; doi:10.1007/s12028-022-01534-y)
Supplement: Supplementary file 1 — (DOCX 378 kb) [file 12028_2022_1534_MOESM1_ESM.docx]

**Table S1: Patient characteristics and univariate analysis for 30-day mortality**

| Patient characteristics | survived (n=209) | deceased (n=73) | p-value |
| --- | --- | --- | --- |
| Age [a] | 55.7 ± 13.1 | 60.5 ± 14.8 | 0.016 |
| Hunt&Hess 1°  2°  3°  4°  5° | 28 (13%)  41 (20%)  47 (23%)  44 (21%)  49 (23%) | 4 (6%)  5 (7%)  9 (12%)  14 (19%)  41 (56%) | < 0.001 |
| sex female:  male: | 148 (71%)  61 (29%) | 45 (62%)  28 (38%) | 0.147 |
| length of ventilation [d] | 15.1 ± 12.4 | 12.1 ± 8.2 | 0.022 |
| on day 1: |  |  |  |
| paO_2_ integral above 80 mmHg [mmHg/d] | 19.5 ± 20.4 | 18.5 ± 15.9 | 0.669 |
| paO_2_ integral above 100 mmHg [mmHg/d] | 9.8 ± 16.0 | 8.5 ± 11.7 | 0.461 |
| paO_2_ integral above 120 mmHg [mmHg/d] | 5.5 ± 12.5 | 4.4 ± 8.7 | 0.410 |
| paO_2_ integral above 150 mmHg [mmHg/d] | 2.8 ± 9.0 | 2.3 ± 6.1 | 0.579 |
| max paO_2_ [mmHg] | 157.4 ± 87.8 | 173.5 ± 120.7 | 0.298 |
| mean paO_2_ [mmHg] | 96.7 ± 22.6 | 95.8 ± 18.1 | 0.744 |
| admission to day 3: |  |  |  |
| paO_2_ integral above 80 mmHg [mmHg/d] | 12.1 ± 11.5 | 13.2 ± 10.4 | 0.481 |
| paO_2_ integral above 100 mmHg [mmHg/d] | 4.9 ± 7.5 | 5.1 ± 6.1 | 0.792 |
| paO_2_ integral above 120 mmHg [mmHg/d] | 2.5 ± 5.4 | 2.6 ± 4.2 | 0.971 |
| paO_2_ integral above 150 mmHg [mmHg/d] | 1.3 ± 3.6 | 1.5 ± 3.2 | 0.622 |
| max paO_2_ [mmHg] | 174.2 ± 101.1 | 201.5 ± 133.0 | 0.113 |
| mean paO_2_ [mmHg] | 88.3 ± 14.2 | 89.8 ± 12.7 | 0.370 |
| admission to day 14: |  |  |  |
| paO_2_ integral above 80 mmHg [mmHg/d] | 9.4 ± 7.4 | 16.1 ± 24.2 | 0.022 |
| paO_2_ integral above 100 mmHg [mmHg/d] | 2.7 ± 3.1 | 9.1 ± 20.7 | 0.011 |
| paO_2_ integral above 120 mmHg [mmHg/d] | 1.1 ± 1.6 | 6.5 ± 17.8 | 0.012 |
| paO_2_ integral above 150 mmHg [mmHg/d] | 0.5 ± 1.0 | 4.5 ± 14.3 | 0.020 |
| max paO_2_ [mmHg] | 212.0 ± 117.2 | 281.8 ± 151.2 | < 0.001 |
| mean paO_2_ [mmHg] | 85.8 ± 9.9 | 92.1 ± 26.2 | 0.048 |

paO­_2_: arterial partial pressure of oxygen, data are given as mean ± standard deviation or numbers and percentage in parentheses, as applicable.

**Table S2: Patient characteristics and univariate analysis for outcome at discharge**

| Patient characteristics | favorable (n=75) | unfavorable (n=207) | p-value |
| --- | --- | --- | --- |
| Age [a] | 51.7 ± 12.6 | 58.8 ± 13.6 | < 0.001 |
| Hunt&Hess 1°  2°  3°  4°  5° | 19 (25%)  21 (28%)  16 (21%)  11 (15%)  8 (11%) | 13 (6%)  25 (12%)  40 (19%)  47 (23%)  82 (40%) | < 0.001 |
| sex female:  male: | 51  24 | 142  65 | 0.924 |
| length of ventilation [d] | 4.9 ± 7.1 | 17.7 ± 10.9 | < 0.001 |
| on day 1: |  |  |  |
| paO_2_ integral above 80 mmHg [mmHg/d] | 17.5 ± 14.3 | 19.9 ± 20.8 | 0.292 |
| paO_2_ integral above 100 mmHg [mmHg/d] | 7.7 ± 10.0 | 10.0 ± 16.4 | 0.149 |
| paO_2_ integral above 120 mmHg [mmHg/d] | 3.9 ± 6.6 | 5.7 ± 13.0 | 0.118 |
| paO_2_ integral above 150 mmHg [mmHg/d] | 1.6 ± 3.7 | 3.1 ± 9.4 | 0.054 |
| max paO_2_ [mmHg] | 146.1 ± 76.6 | 167.2 ± 103.6 | 0.066 |
| mean paO_2_ [mmHg] | 95.2 ± 16.2 | 96.9 ± 23.2 | 0.492 |
| admission to day 3: |  |  |  |
| paO_2_ integral above 80 mmHg [mmHg/d] | 11.2 ± 9.7 | 12.8 ± 11.7 | 0.243 |
| paO_2_ integral above 100 mmHg [mmHg/d] | 4.2 ± 6.0 | 5.2 ± 7.5 | 0.236 |
| paO_2_ integral above 120 mmHg [mmHg/d] | 2.1 ± 4.0 | 2.7 ± 5.4 | 0.290 |
| paO_2_ integral above 150 mmHg [mmHg/d] | 0.9 ± 2.4 | 1.5 ± 3.8 | 0.152 |
| max paO_2_ [mmHg] | 166.3 ± 99.3 | 186.7 ± 114.2 | 0.145 |
| mean paO_2_ [mmHg] | 87.2 ± 12.2 | 89.2 ± 14.3 | 0.247 |
| admission to day 14: |  |  |  |
| paO_2_ integral above 80 mmHg [mmHg/d] | 9.1 ± 7.0 | 11.9 ± 15.9 | 0.042 |
| paO_2_ integral above 100 mmHg [mmHg/d] | 2.4 ± 3.0 | 5.0 ± 12.9 | 0.007 |
| paO_2_ integral above 120 mmHg [mmHg/d] | 0.9 ± 1.4 | 3.0 ± 10.9 | 0.006 |
| paO_2_ integral above 150 mmHg [mmHg/d] | 0.4 ± 0.9 | 2.0 ± 8.7 | 0.009 |
| max paO_2_ [mmHg] | 193.2 ± 110.5 | 243.4 ± 134.5 | 0.002 |
| mean paO_2_ [mmHg] | 85.3 ± 9.6 | 88.1 ± 17.8 | 0.099 |

paO_2_: arterial partial pressure of oxygen, data are given as mean ± standard deviation or numbers and percentage in parentheses, as applicable.

**Table S3: Patient characteristics and univariate analysis for outcome at three months**

| Patient characteristics | favorable (n=110) | unfavorable (n=150) | p-value |
| --- | --- | --- | --- |
| Age [a] | 52.8 ± 12.0 | 60.5 ± 13.4 | < 0.001 |
| Hunt&Hess 1°  2°  3°  4°  5° | 22 (20%)  28 (25%)  25 (23%)  18 (16%)  17 (16%) | 9 (6%)  14 (9%)  26 (17%)  35 (23%)  66 (44%) | < 0.001 |
| sex female:  male: | 78 (71%)  32 (29%) | 106 (71%)  44 (29%) | 0.966 |
| length of ventilation [d] | 9.2 ± 10.1 | 17.7± 11.1 | < 0.001 |
| on day 1: |  |  |  |
| paO_2_ integral above 80 mmHg [mmHg/d] | 19.6 ± 19.1 | 19.9 ± 20.0 | 0.880 |
| paO_2_ integral above 100 mmHg [mmHg/d] | 9.4 ± 14.9 | 10.0 ± 15.5 | 0.757 |
| paO_2_ integral above 120 mmHg [mmHg/d] | 5.2 ± 11.1 | 5.6 ± 12.5 | 0.824 |
| paO_2_ integral above 150 mmHg [mmHg/d] | 2.5 ± 6.8 | 3.0 ± 9.6 | 0.626 |
| max paO_2_ [mmHg] | 151.3 ± 81.1 | 172.1 ± 110.1 | 0.082 |
| mean paO_2_ [mmHg] | 97.1 ± 20.9 | 97.1 ± 22.3 | 0.993 |
| admission to day 3: |  |  |  |
| paO_2_ integral above 80 mmHg [mmHg/d] | 12.4 ± 12.1 | 12.8 ± 10.9 | 0.755 |
| paO_2_ integral above 100 mmHg [mmHg/d] | 5.0 ± 8.1 | 5.2 ± 6.7 | 0.902 |
| paO_2_ integral above 120 mmHg [mmHg/d] | 2.7 ± 5.8 | 2.6 ± 4.8 | 0.894 |
| paO_2_ integral above 150 mmHg [mmHg/d] | 1.3 ± 3.6 | 1.4 ± 3.7 | 0.840 |
| max paO_2_ [mmHg] | 173.0 ± 103.3 | 190.8 ± 118.8 | 0.201 |
| mean paO_2_ [mmHg] | 88.3 ± 14.5 | 89.4 ± 13.6 | 0.535 |
| admission to day 14: |  |  |  |
| paO_2_ integral above 80 mmHg [mmHg/d] | 9.1 ± 7.4 | 13.0 ± 17.8 | 0.017 |
| paO_2_ integral above 100 mmHg [mmHg/d] | 2.6 ± 3.2 | 5.9 ± 14.9 | 0.009 |
| paO_2_ integral above 120 mmHg [mmHg/d] | 1.0 ± 1.7 | 3.8 ± 12.9 | 0.011 |
| paO_2_ integral above 150 mmHg [mmHg/d] | 0.5 ± 1.1 | 2.5 ± 10.2 | 0.017 |
| max paO_2_ [mmHg] | 202.7 ± 119.6 | 255.0 ± 137.5 | 0.001 |
| mean paO_2_ [mmHg] | 85.3 ± 9.9 | 89.4 ± 19.6 | 0.031 |

paO_2_: arterial partial pressure of oxygen, data are given as mean ± standard deviation or numbers and percentage in parentheses, as applicable.

**Table S4: Patient characteristics and univariate analysis for delayed cerebral ischemia**

| Patient characteristics | yes (n=126) | no (n=156) | p-value |
| --- | --- | --- | --- |
| Age [a] | 56.2 ± 14.1 | 57.4 ± 13.4 | 0.457 |
| Hunt&Hess 1°  2°  3°  4°  5° | 7 (6%)  19 (15%)  20 (16%)  32 (25%)  48 (38%) | 25 (16%)  27 (17%)  36 (23%)  26 (17%)  42 (27%) | 0.007 |
| Fisher 1°  2°  3°  4°  missing: | 5 (4%)  2 (2%)  17 (13%)  102 (81%)  0 | 4 (3%)  11 (7%)  22 (14%)  118 (76%)  1 | 0.157 |
| sex female:  male: | 80 (63%)  46 (27%) | 113 (72%)  43 (28%) | 0.108 |
| length of ventilation [d] | 17.4 ± 11.0 | 11.8 ± 11.3 | < 0.001 |
| on day 1: |  |  |  |
| paO_2_ integral above 80 mmHg [mmHg/d] | 19.0 ± 18.1 | 19.5 ± 20.3 | 0.838 |
| paO_2_ integral above 100 mmHg [mmHg/d] | 9.1 ± 13.3 | 9.7 ± 16.3 | 0.764 |
| paO_2_ integral above 120 mmHg [mmHg/d] | 4.9 ± 9.5 | 5.6 ± 13.2 | 0.610 |
| paO_2_ integral above 150 mmHg [mmHg/d] | 2.2 ± 6.0 | 3.1 ± 9.8 | 0.340 |
| max paO_2_ [mmHg] | 155.7 ± 76.8 | 166.4 ± 111.4 | 0.339 |
| mean paO_2_ [mmHg] | 96.4 ± 20.2 | 99.5 ± 22.6 | 0.971 |
| admission to day 3: |  |  |  |
| paO_2_ integral above 80 mmHg [mmHg/d] | 12.6 ± 11.0 | 12.2 ± 11.4 | 0.806 |
| paO_2_ integral above 100 mmHg [mmHg/d] | 4.9 ± 6.9 | 5.0 ± 7.4 | 0.952 |
| paO_2_ integral above 120 mmHg [mmHg/d] | 2.5 ± 4.7 | 2.6 ± 5.4 | 0.819 |
| paO_2_ integral above 150 mmHg [mmHg/d] | 1.2 ± 3.1 | 1.4 ± 3.8 | 0.666 |
| max paO_2_ [mmHg] | 174.9 ± 94.9 | 186.4 ± 122.0 | 0.373 |
| mean paO_2_ [mmHg] | 89.2 ± 13.4 | 88.2 ± 14.1 | 0.523 |
| admission to day 14: |  |  |  |
| paO_2_ integral above 80 mmHg [mmHg/d] | 11.2 ± 10.2 | 11.1 ± 16.6 | 0.922 |
| paO_2_ integral above 100 mmHg [mmHg/d] | 4.1 ± 7.1 | 4.5 ± 13.6 | 0.707 |
| paO_2_ integral above 120 mmHg [mmHg/d] | 2.3 ± 5.8 | 2.7 ± 11.6 | 0.712 |
| paO_2_ integral above 150 mmHg [mmHg/d] | 1.4 ± 4.4 | 1.7 ± 9.3 | 0.710 |
| max paO_2_ [mmHg] | 247.9 ± 129.4 | 215.6 ± 129.6 | 0.038 |
| mean paO_2_ [mmHg] | 88.1 ± 12.0 | 86.8 ± 18.7 | 0.514 |

paO_2_: arterial partial pressure of oxygen, data are given as mean ± standard deviation or numbers and percentage in parentheses, as applicable.

**Table S5: Multivariable analysis – 30-day mortality**

| **Parameter** | **Odds Ratio** | **lower CI** | **upper CI** | **p-value** |
| --- | --- | --- | --- | --- |
| Integral above 80 mmHg | 1.066 | 1.026 | 1.108 | 0.001 |
| Integral above 100 mmHg | 1.164 | 1.069 | 1.268 | < 0.001 |
| Integral above 120 mmHg | 1.358 | 1.149 | 1.606 | < 0.001 |
| Integral above 150 mmHg | 1.590 | 1.216 | 2.078 | 0.001 |
| Maximum paO_2_ | 1.005 | 1.003 | 1.008 | < 0.001 |
| Mean paO_2_ | 1.042 | 1.014 | 1.071 | 0.003 |

Integrals after 14 days; paO­_2_: arterial partial pressure of oxygen, CI: 95% confidence intervals. Separate logistic-regression model for each oxygenation parameter adjusted for age, Hunt&Hess, sex, and duration of mechanical ventilation.

**Table S6: Multivariable analysis – favorable outcome at discharge**

| **Parameter** | **Odds Ratio** | **lower CI** | **upper CI** | **p-value** |
| --- | --- | --- | --- | --- |
| Integral above 80 mmHg | 0.973 | 0.936 | 1.012 | 0.170 |
| Integral above 100 mmHg | 0.929 | 0.853 | 1.012 | 0.091 |
| Integral above 120 mmHg | 0.853 | 0.711 | 1.024 | 0.088 |
| Integral above 150 mmHg | 0.775 | 0.570 | 1.054 | 0.105 |
| Maximum paO_2_ | 0.998 | 0.995 | 1.001 | 0.185 |
| Mean paO_2_ | 0.986 | 0.960 | 1.013 | 0.322 |

Integrals after 14 days; paO­_2_: arterial partial pressure of oxygen, CI: 95% confidence intervals. Separate logistic-regression model for each oxygenation parameter adjusted for age, Hunt&Hess, sex, and duration of mechanical ventilation.

**Table S7: Multivariable analysis – favorable outcome after three months**

| **Parameter** | **Odds Ratio** | **lower CI** | **upper CI** | **p-value** |
| --- | --- | --- | --- | --- |
| Integral above 80 mmHg | 0.955 | 0.920 | 0.991 | 0.015 |
| Integral above 100 mmHg | 0.904 | 0.836 | 0.978 | 0.012 |
| Integral above 120 mmHg | 0.832 | 0.716 | 0.968 | 0.017 |
| Integral above 150 mmHg | 0.769 | 0.611 | 0.969 | 0.026 |
| Maximum paO_2_ | 0.997 | 0.995 | 0.999 | 0.012 |
| Mean paO_2_ | 0.969 | 0.943 | 0.996 | 0.026 |

Integrals after 14 days; paO­_2_: arterial partial pressure of oxygen, CI: 95% confidence intervals. Separate logistic-regression model for each oxygenation parameter adjusted for age, Hunt&Hess, sex, and duration of mechanical ventilation.

**Table S8: Multivariable analysis – delayed cerebral ischemia**

| **Parameter** | **Odds Ratio** | **lower CI** | **upper CI** | **p-value** |
| --- | --- | --- | --- | --- |
| Integral above 80 mmHg | 1.000 | 0.982 | 1.018 | 0.993 |
| Integral above 100 mmHg | 0.997 | 0.974 | 1.021 | 0.822 |
| Integral above 120 mmHg | 0.998 | 0.971 | 1.025 | 0.866 |
| Integral above 150 mmHg | 0.997 | 0.963 | 1.032 | 0.859 |
| Maximum paO_2_ | 1.001 | 0.999 | 1.003 | 0.157 |
| Mean paO_2_ | 1.003 | 0.987 | 1.019 | 0.703 |

Integrals after 14 days; paO­_2_: arterial partial pressure of oxygen, CI: 95% confidence intervals. Separate logistic-regression model for each oxygenation parameter adjusted for age, Hunt&Hess, sex, and duration of mechanical ventilation.

**Table S9: Time-weighted mean inspiratory fraction of oxygen after 14 days**

| **Parameter** | **Q1 (62-78 mmHg)** | **Q2 (78-85 mmHg)** | **Q3 (85-93 mmHg)** | **Q4 (93-228 mmHg)** |
| --- | --- | --- | --- | --- |
| FiO_2_ | 0.38 ± 0.15 | 0.34 ± 0.13 | 0.33 ± 0.11 | 0.35 ± 0.13 |

FiO_2_: inspiratory fraction of oxygen; Q1 to Q4 refer the quartiles obtained by categorization according to time-weighted mean arterial oxygen partial pressure after 14 days; data are given as mean ± standard deviation.

No difference was found by Analysis of Variance (ANOVA), p=0.131. Post-Hoc tests for multiple comparisons with Bonferroni correction showed no statistically significant difference between groups.

**Table S10: Discontinuation of mechanical ventilation before day 14**

| **Parameter** | **Q1 (62-78 mmHg) n=71** | **Q2 (78-85 mmHg) n=70** | **Q3 (85-93 mmHg) n=70** | **Q4 (93-228 mmHg) n=71** | **p-Value** |
| --- | --- | --- | --- | --- | --- |
| Extubation | 26 (37%) | 27 (39%) | 25 (36%) | 13 (18%) | 0.035 |
| Death | 14 (20%) | 13 (19%) | 7 (10%) | 18 (25%) | 0.130 |
| Extubation or death | 40 (56%) | 40 (57%) | 32 (46%) | 31 (44%) | 0.244 |

Q1 to Q4 refer the quartiles obtained by categorization according to time-weighted mean arterial oxygen partial pressure after 14 days; separate Chi-square tests were done for each parameter; data are given as numbers and percent in parentheses. Table only includes deaths up to day 14.

**Table S11: Time-weighted mean oxygen partial pressures before and after extubation**

| **Parameter** | **before extubation** | **after extubation** | **p-Value** |
| --- | --- | --- | --- |
| Mean paO_2_ [mmHg] | 85.7 ± 13.4 | 86.5 ± 36.9 | 0.765 |

Patients extubated before day 14 included in analysis (n=127); paO_2_: arterial partial pressure of oxygen.

**Table S12: Thirty-day mortality – cause of death**

|  | **Q1 (62-78 mmHg) n=71** | **Q2 (78-85 mmHg) n=70** | **Q3 (85-93 mmHg) n=70** | **Q4 (93-228 mmHg) n=71** |
| --- | --- | --- | --- | --- |
| unrelated to SAH | 2 | 1 | 3 | 0 |
| withdrawal of care due to poor prognosis and presumed will of patient | 15 | 10 | 9 | 8 |
| brain death | 3 | 3 | 4 | 15 |
| total | 20 | 14 | 16 | 23 |

SAH: subarachnoid hemorrhage; Q1 to Q4 refer the quartiles obtained by categorization according to time-weighted mean arterial oxygen partial pressure after 14 days; Chi-square reveals statistically significant difference, p=0.008; data are given as numbers. Table includes deaths up to day 30.


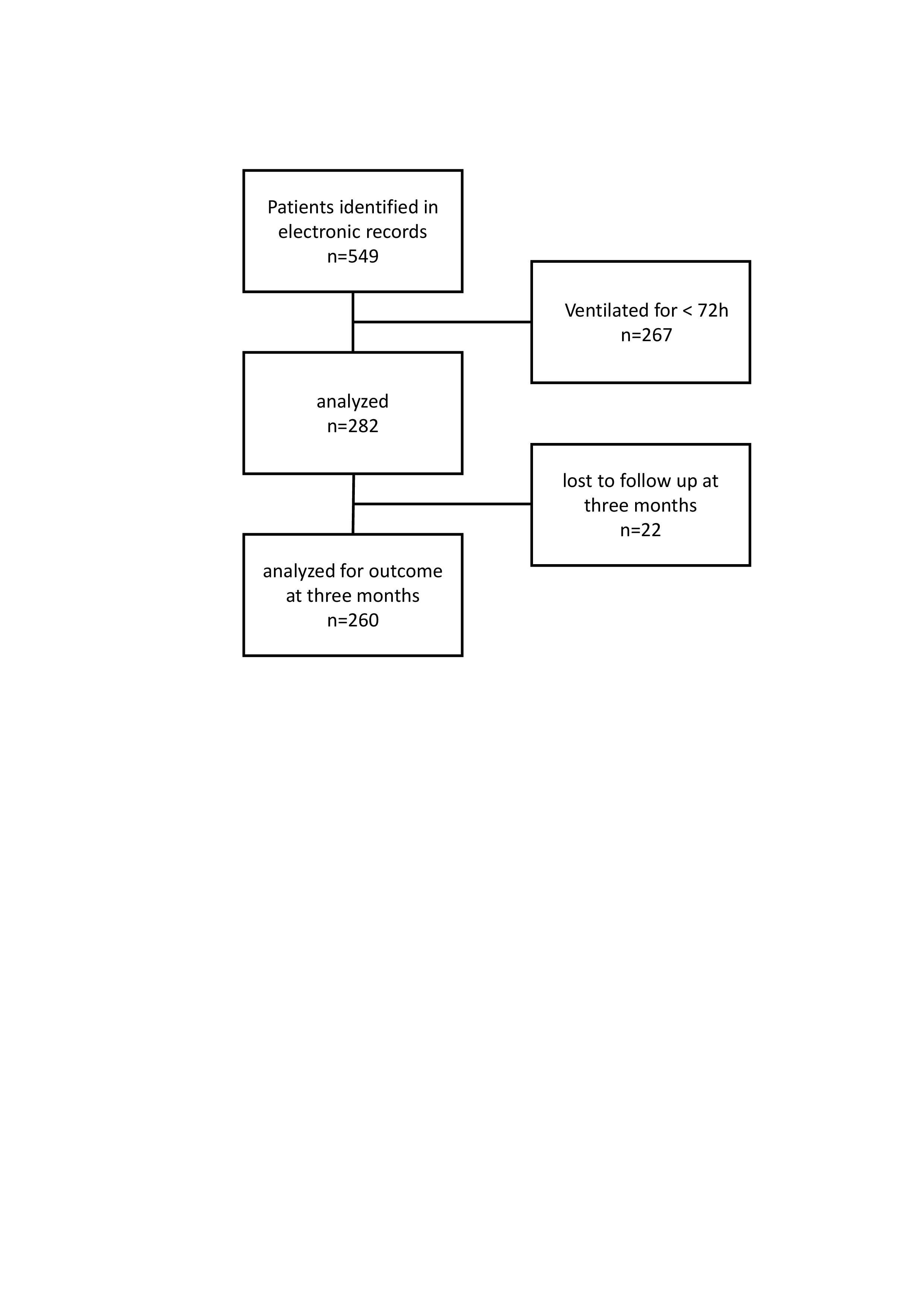


**Figure S1: Patient inclusion**


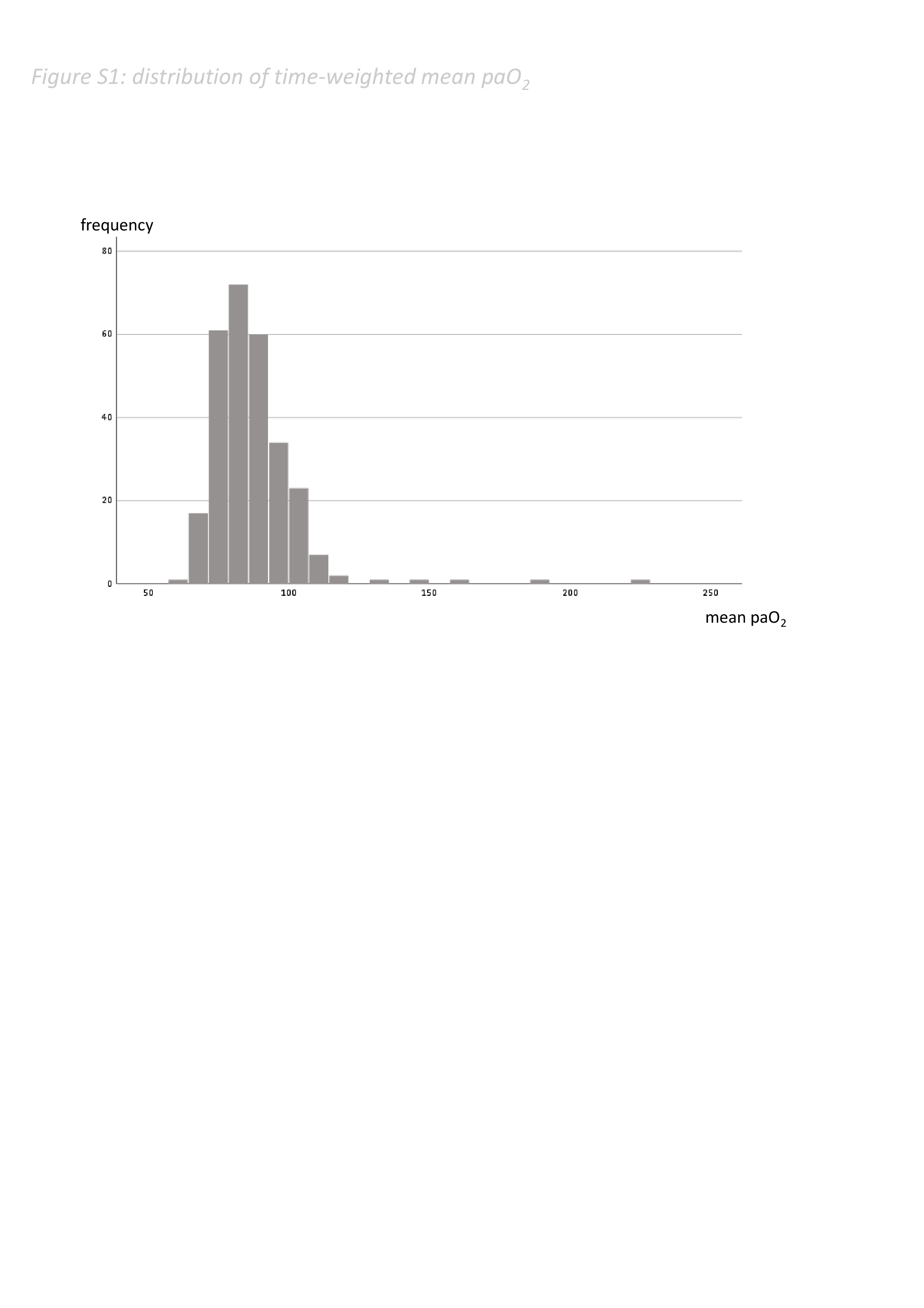


**Figure S2: Distribution of time-weighted mean arterial partial pressure of oxygen after 14 days**

paO_2_: arterial partial pressure of oxygen, minimum 62mmHg, 1^st^ quartile: 78mmHg, median: 85mmHg, 3^rd^ quartile: 93mmHg, maximum: 228mmHg.


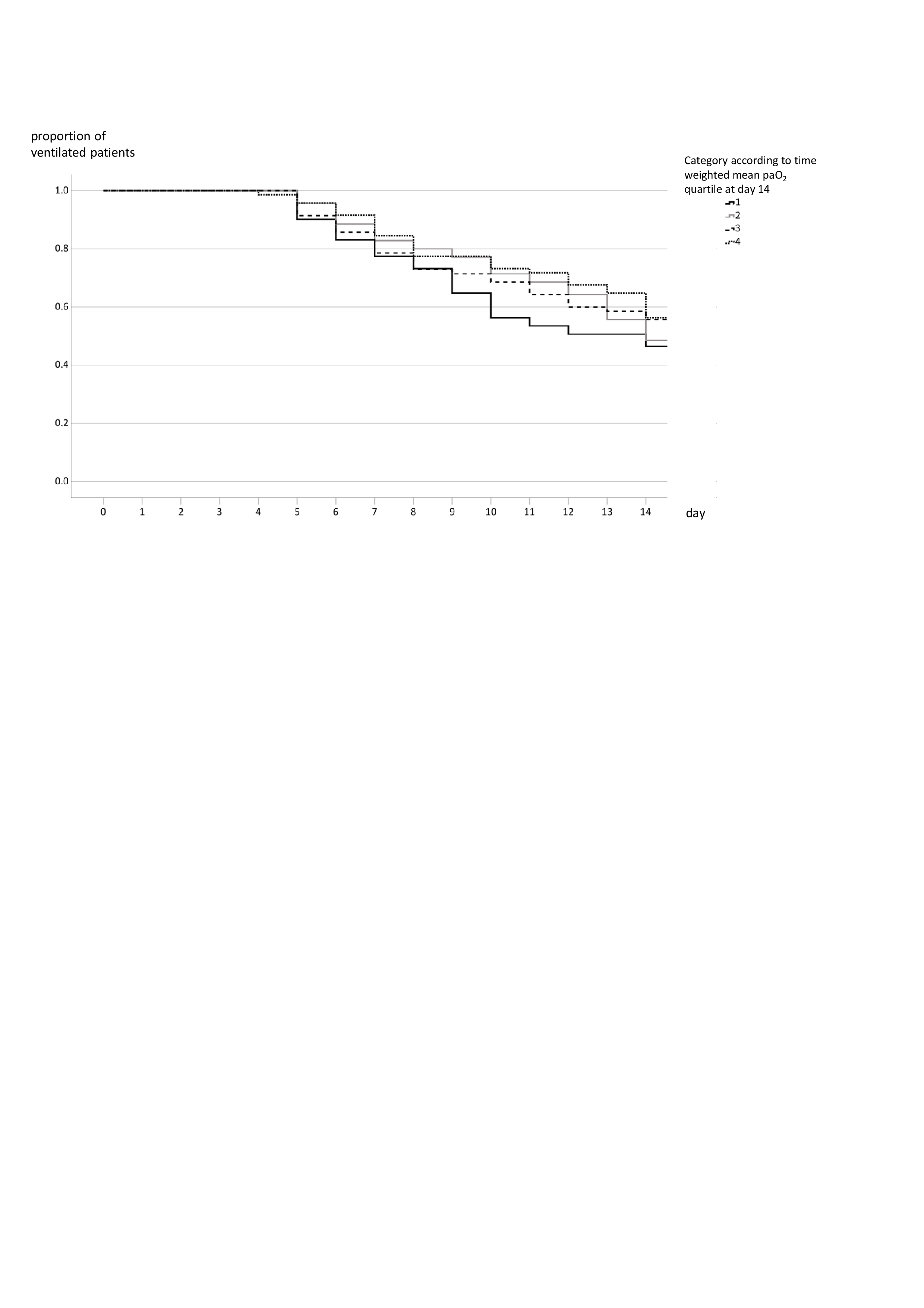


**Figure S3: Proportion of mechanically ventilated patients over time**

No significant difference could be demonstrated between groups categorized by time-weighted mean arterial oxygen partial pressure after 14 days (Mantel-Cox log-rank test, p=0.302). Quartile 1: 62 to 78mmHg, quartile 2: 78 to 85mmHg, quartile 3: 85 to 93mmHg, quartile 4: 93 to 228mmHg.
